# Effects of Docosahexaenoic Supplementation and *In Vitro* Vitamin C on the Oxidative and Inflammatory Neutrophil Response to Activation

**DOI:** 10.1155/2015/187849

**Published:** 2015-04-19

**Authors:** Xavier Capó, Miquel Martorell, Antoni Sureda, Josep Antoni Tur, Antoni Pons

**Affiliations:** ^1^Research Group on Community Nutrition and Oxidative Stress, Science Laboratory of Physical Activity, Department of Fundamental Biology and Health Sciences, University of Balearic Islands, 07122 Palma de Mallorca, Spain; ^2^CIBER: CB12/03/30038 Fisiopatología de la Obesidad y la Nutrición, CIBEROBN, Instituto de Salud Carlos III (ISCIII), University of Balearic Islands, 07122 Palma de Mallorca, Spain; ^3^Departamento de Nutrición y Dietética, Facultad de Farmacia, Universidad de Concepción, 4070386 Concepción, Chile

## Abstract

We studied the effects of diet supplementation with docosahexaenoic (DHA) and* in vitro* vitamin C (VitC) at physiological concentrations on oxidative and inflammatory neutrophil response to phorbol myristate acetate (PMA). Fifteen male footballers ingested a beverage enriched with DHA or a placebo for 8 weeks in a randomized double-blind study. Neutrophils were isolated from blood samples collected in basal conditions at the end of nutritional intervention. Neutrophils were cultured for 2 hours at 37°C in (a) control media, (b) media with PMA, and (c) media with PMA + VitC. PMA induces neutrophil degranulation with increased extracellular myeloperoxidase and catalase activities, nitric oxide production, expression of the inflammatory genes cyclooxygenase-2, nuclear factor *κβ*, interleukin 8 and tumor necrosis factor *α*, and interleukin 6 production. DHA diet supplementation boosts the exit of CAT from neutrophils but moderates the degranulation of myeloperoxidase granules induced by PMA. VitC facilitates azurophilic degranulation of neutrophils and increases gene expression of myeloperoxidase induced by PMA. VitC and DHA diet supplementation prevent PMA effects on inflammatory gene expression, although together they do not produce additional effects. DHA diet supplementation enhances antioxidant defences and anti-inflammatory neutrophil response to* in vitro* PMA activation. VitC facilitates neutrophil degranulation but prevents an inflammatory response to PMA.

## 1. Introduction

Neutrophils, along with other granulocytes, play an important role in inflammation both for clearing pathogens and for immune regulation [1] and also participate in remodelling damaged tissue. Neutrophils are recruited to the site of injury or infection where they can trigger an inflammatory response by producing cytokines or other chemical mediators and antimicrobial agents to deal with possible infection or contribute to tissue repair [2]. The phagocytic neutrophil function involves an oxidative burst carried out by the enzyme NADPH-oxidase and the secretion of lysosomal enzymes such as proteases, phospholipases, and glycosidases among others [3].

Exercise produces an acute phase immune response characterised by neutrophilia and lymphopenia [4, 5], thus altering the lymphocyte and neutrophil oxidative balance [6]. Acute exercise primes neutrophils to oxidative burst [7], and it significantly increases neutrophil ROS production after stimulation with zymosan or phorbol myristate acetate (PMA) [8]. The priming of neutrophils induced by acute exercise is in parallel with neutrophil secretion of CAT [7]. Extracellular CAT regulates extracellular hydrogen peroxide levels, probably to minimize oxidative damage in plasma and also to end the signalling action of the hydrogen peroxide [8]. Low levels of antioxidant enzymes in neutrophils after exercise enhance the importance of low molecular weight antioxidant molecules in order to prevent oxidative damage. Ascorbate is found in normal circulating human neutrophils in millimolar concentration [6, 9] and its levels duplicate in neutrophils after prolonged acute exercise [6]. The contribution of ascorbate is a key element to neutrophil protection against oxidative imbalance after acute exercise [6, 10]. However, the influence of ascorbate on neutrophil activation and on its immune function has been little studied [11].

Ascorbate influences some neutrophil functions, including augmented chemotaxis, increased particulate ingestion, enhanced lysozyme-mediated nonoxidative killing, and protection against the toxic effects of superoxide anion radical [11]. Some studies on humans have shown that high doses of ascorbate (20 mM) can inhibit production of IL6 and TNF*α* in monocytes without affecting IL1 or IL8 levels, but the same ascorbate concentrations have also been found to inhibit IL2 production without affecting TNF*α* or IFN*γ* levels in lymphocytes [12]. It has been indicated that the physiological concentration of ascorbate in neutrophils is about 1–3 mM depending on exercise status [5]. These physiological ascorbate levels are lower than ascorbate concentrations assayed in other studies [12, 13]. The effects of physiological doses of ascorbate after vigorous exercise on neutrophil inflammatory and immune capabilities have not been studied, even though there is evidence that a millimolar physiological concentration of ascorbate could inhibit NF*κβ* activation in endothelial cells [13].

PMA is a molecular mimetic of diacylglycerol [14] used to activate neutrophils in order to mimic neutrophil response to acute exercise [8]. PMA activates several isoforms of protein kinase C, particularly PKC*δ* and *ε*, which in turn activate the NF*κβ* signalling pathway [15–17] in neutrophils [18, 19]. PMA induces neutrophil degranulation and the assembly and activation of lysosomal NADPH-oxidase [20, 21]. Moreover, PMA increases reactive oxygen species [22] production and antioxidant enzyme gene expression in neutrophils [18] and in HL60 cells [8]. The production of ROS, induced by PMA, is attributed to NADPH-oxidase operation in the cell or lysosomal membranes [23], but it is also attributed to the mitochondrial respiratory chain function [8, 24]. Activated neutrophils produce an oxidative burst that in turn can cause oxidative damage in proteins, lipids, or nucleic acid [25]. Similarly, neutrophils may modify the pattern of nitric oxide (NO) availability after intense exercise [10]. Nitric oxide (NO) regulates several important neutrophil functions, including chemotaxis, adhesion, aggregation, and PMN-mediated bacterial killing or tissue damage [26, 27]. NO is synthesized in neutrophils by constitutive and inducible nitric oxide synthase isoforms (iNOS). iNOS in combination with NAD(P)H-oxidase play a role in peroxynitrite production acting as a microbiocide together with other products such as hypochlorous acid, produced by myeloperoxidase [28–30].

The neutrophil response to PMA is affected by omega-3-PUFAs in isolated neutrophils in rats [31]. Omega-3-PUFAs increase phagocytic and antifungal activity without altering nitric oxide production. They increase production of hydrogen peroxide and superoxide anion radical in the presence of PMA but do not modify superoxide anion production in the presence of zymosan [31]. In goats, DHA increases neutrophil defensive functions by upregulating phagocytosis activity and downregulating ROS production, thus reducing excessive tissue damage [32]. In humans, no significant effects on neutrophil chemotaxis or superoxide anion radical production induced by DHA have been reported [33]. Although DHA diet supplementation has little influence on neutrophil capabilities to produce ROS after exercise, it reduces the time at which maximal neutrophil ROS production after stimulation with opsonized zymosan is attained; DHA supplementation induces a faster response to zymosan after exercise in neutrophils [8]. The “*in vitro*” generation of inflammatory precursors by human neutrophils does not seem to be modulated by DHA added in the cell medium [34]. In a similar way, DHA diet supplementation hardly modifies the antioxidant adaptive response of neutrophils to training, and no effects have been reported on the production of reactive oxygen and nitrogen species (ROS and RNS) by neutrophils stimulated with PMA after acute exercise [35], probably attributable to a lack of neutrophil activation after exercise. However, the effects of DHA diet supplementation on neutrophil response against a molecular mimetic of DAG, such as PMA, in trained sportsmen are not known.

The aim of this study was to assess the potential role of dietary supplementation with DHA for 8 weeks in highly trained footballers in the oxidative and inflammatory neutrophil response against their activation “*in vitro*” with PMA and the role of physiological neutrophil ascorbate levels in these immune responses of activated neutrophils.

## 2. Material and Methods

### 2.1. Subjects and Study Design

Neutrophils were obtained from fifteen male professional and federated football players who volunteered to take part in this study (Table 1). The subjects and study design was the same as described previously [35, 36]. It was a double-blind study of eight weeks of nutritional intervention with DHA diet supplementation which was registered at ClinicalTrial.gov (NCT02177383). The participants were 22 football players randomly allocated either to the supplemented (*n* = 11) or to the placebo group (*n* = 11). During the nutritional intervention 2 football players of the experimental group left to train with Mallorca B team and they abandoned the study, and 5 football players of the placebo group dropped out of trial for different reasons, one of them broke the anterior cruciate ligament of knee, 2 football players left the team, and the other two were promoted to Mallorca A team. Each group consumed one litre of DHA-enriched or placebo (DHA-nonsupplemented) drinks five times a week. All subjects were informed of the purpose and demands of the study before giving their written consent to participate. The study protocol was in accordance with the Declaration of Helsinki for research on human subjects and was approved by the Ethical Committee of Clinical Investigation of the Autonomous Community of the Balearic Islands (Palma de Mallorca, Balearic Islands, Spain). Participants in the study were 19.7 ± 0.4 years old, 76.5 ± 2.5 kg in weight, and 179.5 ± 2.5 cm tall. Waist circumference was 78.4 ± 0.9 cm; hip circumference was 97.4 ± 1.2 cm; and waist-hip ratio was 0.81 ± 0.01 WHR. Body mass index was 23.7 ± 0.55 BMI, kg/m^2^. The football players had 92.6 ± 0.2% fat-free mass. VO_2_max, determined following the test of Leger-Boucher [37], was 61.4 ± 1.35 mL/kg min. The study was performed at the beginning of the competitive season. DHA supplementation was administered to the footballers for eight weeks, using an almond-based experimental drink enriched with DHA. In order to avoid beverage influence, DHA-nonsupplemented drink was given to the placebo group. Both experimental and placebo drinks were enriched with the same amount of *α*-tocopherol-acetate. There were no differences between anthropometric characteristics or physical activity capabilities, such as age, body weight, body mass index, waist-hip ratio, percentage of fat mass, percentage of fat-free mass, daily physical activity time, or their performance status measured as the VO_2_max between placebo and experimental groups. Neither were there any significant differences found between the number of circulating neutrophils in the experimental and placebo group [35].

### 2.2. Drinks Composition and Diet Supplementation

Both the placebo and the experimental drinks contained 3.0% almond, 0.8% sucrose, and 0.8% different lipids, depending on the type of drink: traces of lemon and cinnamon flavours and 40 mg/1L of vitamin E (*α*-tocopherol-acetate). The lipid content in the placebo drink was 0.8% refined olive oil whereas in the experimental drink it was 0.6% of the same refined olive oil and 0.2% DHA-S Market (Martek Biosciences Corporation, Columbia, EEUU). The two almond drinks were produced by Liquats Vegetals S.A. (Girona, Spain) following standardized procedures [35, 36]. The placebo and experimental beverages were identical in both taste and appearance.

The experimental drink had significantly higher concentrations of the fatty acids C20:3 (21 *μ*M), C22:0 (76 *μ*M), C22:5 (1715 *μ*M), and C22:6n3 (3457 *μ*M) than the placebo drink in which they were undetected. The daily intake of 1 litre of experimental beverage for five days a week during eight weeks represented a mean daily supplementation intake of 1.14 g of DHA to which the intake of omega-3 in the diet must be added. DHA intake of the placebo group was only from the diet. Nutrient intake by the diet was determined beforehand using a previously published seven-day questionnaire [35].

### 2.3. Assessment of Nutritional Intervention

The impact of DHA diet supplementation was measured by determining fatty acid composition of erythrocyte membranes before and after nutritional intervention. Erythrocytes were obtained from the blood samples taken at the beginning and at the end of the nutritional intervention as indicated above. Fatty acids were extracted [38] and analysed following a previously described procedure [35].

### 2.4. Neutrophil Purification

Venous blood samples were obtained from the antecubital vein of sportsmen in suitable vacutainers with EDTA as anticoagulant. Blood samples were obtained after eight weeks of nutritional intervention at 08:00 on a training day after 12 h overnight fasting. Neutrophil fraction was purified following an adaptation of the method described by Bøyum [39]. Blood was carefully introduced on Ficoll in a proportion of 1.5 : 1 and was then centrifuged at 900 g, at 4°C for 30 min. The precipitate containing the erythrocytes and neutrophils was incubated at 4°C with 0.15 M ammonium chloride to haemolyse erythrocytes. The suspension was centrifuged at 750 g, at 4°C for 15 min, and the supernatant was then discarded. The neutrophil phase at the bottom was washed first with ammonium chloride and then with phosphate buffer saline (PBS), pH 7.4.

### 2.5. Cell Culture

Purified neutrophils from experimental and placebo groups were divided into three aliquots, each of which was cultured with RPMI 1640 culture medium containing 2 mM L-glutamine, but three different treatments were applied: control group: neutrophils treated only with culture medium (RPMI 1640); PMA group: neutrophils treated with culture medium (RPMI 1640) in addition to PMA (PMA 5 *μ*g/mL); PMA and vitamin C group: neutrophils treated with culture medium (RPMI 1640) in addition to PMA (PMA 5 *μ*g/mL) and ascorbate (3 mM). All neutrophil groups were incubated in polypropylene tubes at 37°C for 2 hours. Subsequently, the cells were pelleted by centrifugation (900 ×g, 5 min, 4°C) and cell-free supernatants were stored at −80°C until biochemical determinations; the determinations made in the cell-free supernatants will be considered as determinations in the extracellular media. Neutrophils were resuspended with 2 mL of PBS and divided into two aliquots of 1 mL. One aliquot was centrifuged 900 ×g, 5 min, 4°C, and the precipitate containing the neutrophils was lysed with distilled water and stored at −80°C; determinations performed in the neutrophils lysates will be considered as determinations in the intracellular media. A second aliquot was centrifuged 900 ×g, 5 min, 4°C, and the neutrophil phase at the bottom was used to obtain RNA by adding 1 mL of Tripure and storing at −80°C until further processing.

### 2.6. Enzymatic Determinations

The activities of CAT and MPO were determined both in the lysed neutrophil solution (intracellular media) and in the cell-free culture supernatant (extracellular media). Both enzyme activities were determined with a Shimadzu UV-2100 spectrophotometer at 37°C. MPO activity was measured by guaiacol oxidation [40]. The reaction mixture contained sodium phosphate buffer pH 7 and 13.5 mM guaiacol. The reaction was initiated by adding 300 mM H_2_O_2_, and changes at 470 nm were monitored. CAT activity was measured by the spectrophotometric method of Aebi [41] based on following the decomposition of H_2_O_2_ at 240 nm.

### 2.7. Cytokine Determination

Cytokine (IL6 and TNF*α*) determinations were performed in neutrophil-free supernatant using individual ELISA kits (Diaclone, lit for GEN-PROBE) following the manufacturer's instructions for use. The overall intra-assay coefficient of variation was calculated to be 3.3% for TNF*α* and 4.4% for IL6; the calculated overall interassay coefficient of variation was 9.0% for TNF*α* and 9.1% for IL6.

### 2.8. Nitrite and Nitrate Determination

We centrifuged the neutrophil lysates at 900 ×g at 4°C for 10 min to eliminate cellular debris. The resulting supernatant and the cell-free supernatants were used to measure nitrite and nitrate levels by its transformation into NO which was detected by gas-phase chemiluminescence reaction with ozone using a nitric oxide analyzer (NOA) 280i (Sievers). Nitrite levels were determined following an adaptation of the method described by Castegnaro et al. [42]. Briefly, the purge vessel was loaded with 50 mM KCI in glacial acetic acid and 400 *μ*L of antifoam. A nitrite standard (0.5–10 *μ*M) was used to calculate nitrite concentration. 100 *μ*L of sample or standard was injected in the purge vessel and the area under the curve of NO peaks was recorded and processed using Liquid software. Nitrate levels were determined following an adaptation of the method described by Braman and Hendrix [43]. The purge vessel was loaded with a saturated VCl_3_ solution in 1 M HCl and tempered to 90°C with a current of hot water. To prevent damage to the NOA from the hydrochloric acid vapour, a gas bubbler filled with 1 M NaOH was installed between the purge vessel and the NOA. A nitrate standard (5–200 *μ*M) was used to calculate nitrate concentration. 10 *μ*L of sample or standard was injected in the purge vessel and the area under the curve of NO peaks was recorded and processed using Liquid software. NO_*x*_ was calculated by adding the extracellular and intracellular concentrations of nitrates and nitrites. To calculate intracellular nitrate concentration it was considered a neutrophil volume of 30 × 10^−8^ *μ*L/neutrophil [44].

### 2.9. Neutrophil RNA Extraction and Relative Quantitative RT-PCR Assay

Total RNA was isolated from neutrophils by Tripure extraction (Roche Diagnostics, Germany). RNA (1 *μ*g) from each sample was reverse-transcribed using 50 U of Expand Reverse Transcriptase (Roche Diagnostics, Germany) and 20 pmol oligo [45] for 60 min at 37°C in a 10 *μ*L final volume. The resulting cDNA (2.5 *μ*L) was amplified using the LightCycler FastStart DNA MasterPLUS SYBR Green I kit (Roche Diagnostics, Germany). COX2 TNF*α*, IL8 NF*κβ*, and MPO mRNA expression were determined by multiplex real time rtPCR using human 18S rRNA as invariant reference. The primers and amplification conditions used are listed in Table 2. Relative quantification was performed by standard calculations considering 2^(ΔΔCt)^. mRNA levels from the control were arbitrarily referred to as 1.

### 2.10. Statistical Analysis

Statistical analysis was carried out using the Statistical Package for Social Sciences (SPSS) v.18.0 for Windows. Results are expressed as mean ± SEM. And *p* < 0.05 was considered statistically significant. A Kolmogorov-Smirnov test was applied to assess the normal distribution of the data. The statistical significance of the data was assessed by two-way analysis of variance (ANOVA). The statistical factors analysed were beverage supplementation (S) and neutrophil treatment (T). The sets of data in which there was a significant SxE interaction were tested by one-way ANOVA. When significant effects of S or E factor were found, Student's *t*-test for unpaired data was used to determine the differences between the groups involved.

## 3. Results

### 3.1. Assessment of Nutritional Intervention by Fatty Acid Erythrocyte Composition

DHA supplementation with the experimental beverage significantly increased the levels of this fatty acid in the erythrocyte membranes. At the beginning of the nutritional intervention there were no differences in the fatty acid composition of erythrocyte membranes between the placebo (DHA concentration is 29.0 ± 1.3 nmol/10^9^ erythrocytes) and experimental groups (DHA concentration is 34.0 ± 3.6 nmol/10^9^ erythrocytes). After eight weeks of nutritional intervention with DHA-enriched or non-DHA-enriched drinks the DHA concentration in erythrocytes of the placebo group was 33.6 ± 3.16 nmol/10^9^ erythrocytes and 43.0 ± 3.66 nmol/10^9^ erythrocytes in the supplemented group. Nutritional intervention increased DHA levels 26.5% with respect to initial values in erythrocytes from the experimental group, whereas its levels were unchanged in the placebo group. No effects of either beverage were reported on the amounts of saturated fatty acids (SFAs), monounsaturated fatty acids (MUFAs), or PUFAs on erythrocyte fatty acid composition.

### 3.2. Influence of PMA, Vitamin C, and DHA Diet Supplementation on Neutrophil Oxidative Response

We have studied the influence of PMA, vitamin C, and DHA diet supplementation on neutrophil CAT and MPO activities in lysed neutrophils solution (intracellular media) and in free-cells culture supernatants (extracellular media).

**Figure 1:**
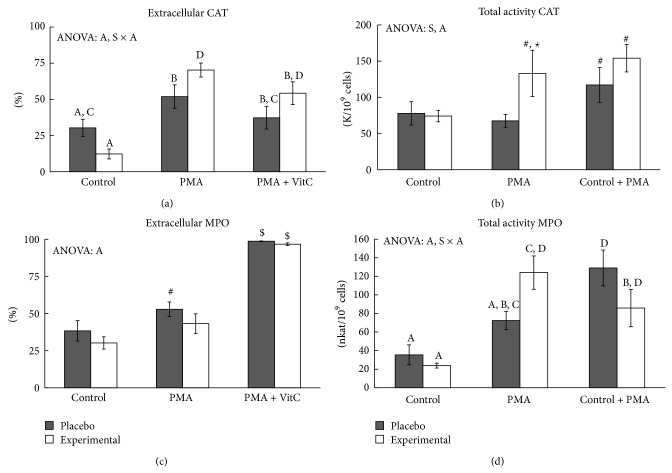
Effects of* in vitro* PMA activation, vitamin C, and DHA diet supplementation on total CAT and MPO enzyme activities and on their distribution in the extracellular compartment of neutrophils. Statistical analysis: two-way ANOVA, *p* < 0.05. S: significant effect of DHA dietary supplementation; A: significant effect of PMA activation; S × A: significant interaction between DHA dietary supplementation and PMA activation effects.** ∗** indicates differences between placebo and experimental groups; # indicates difference with respect to the control group; $ indicates differences between PMA and PMA + VitC group. When interaction S × A exists between supplementation and activation factors, different lowercase letters reveal significant differences. Results are the mean ± SEM.

PMA activation significantly increased total CAT activity in neutrophils (1.8 times) with respect to unstimulated control cells in the experimental group, whereas no differences were reported in the placebo group (Figure 1(a)). These differential responses to PMA resulted in significantly higher total CAT activity in the experimental group when compared to the placebo group. The addition of vitamin C combined with PMA significantly increased total CAT activity both in the placebo and in the experimental groups, without differences between them. Neutrophil activation with PMA, DHA diet supplementation, and vitamin C addition influence extra/intracellular CAT activity distribution, with a significant interaction between these factors on the percentage of neutrophil extracellular CAT activity (Figure 1(b)). The percentage of CAT activity in the extracellular medium significantly increased after neutrophil activation with PMA both in the placebo (1.71 times) and in the experimental (5.71 times) groups. PMA activation produced a significantly higher percentage of CAT activity in the extracellular medium (1.4 times) in the experimental rather than the placebo group. The addition of vitamin C attenuated the effect of PMA activation on the percentage of extracellular enzyme activity, mainly in the placebo group, which returned to control level, whereas the experimental group continued with higher values than the control level. The intra/extracellular distribution of neutrophil CAT activity was significantly altered by PMA activation in both groups (Table 3). Neutrophil CAT release into the extracellular media was reinforced by DHA with a significant increase in CAT activity, about 10 times, in the experimental group, but only about 2.4 times in the placebo group, after PMA stimulation. In fact, extracellular CAT activity in the experimental group was 2.8 times higher than the placebo group after PMA activation. Vitamin C did not modify the CAT activity measured in the extracellular media in PMA-activated neutrophils. These pictures are complementary to those present in the intracellular CAT activity of neutrophils with a decrease about 2.5 after PMA activation, both in the placebo and in the experimental groups. However, the addition of vitamin C together with PMA maintained intracellular CAT activity at control levels.

The possible effect of vitamin C present in the culture medium on the determination of catalase activity was assessed. The presence of vitamin C in the medium used to determine CAT activity could contribute to consumption of hydrogen peroxide, reducing its availability for CAT. The 3 mM concentration of vitamin C added in the neutrophil incubation medium resulted in a concentration of 40 *μ*M in the medium used to determine CAT activity. There was no evidence that the addition of 3 mM vitamin C to the neutrophil incubation medium influenced CAT activity measurement at this concentration of vitamin C.

Neutrophil activation with PMA significantly increased total MPO activity in the experimental group but not in the placebo group, whereas the combined addition of PMA and vitamin C increased total MPO activity in both groups with respect to the control values (Figure 1(c)). The addition of vitamin C to PMA-activated neutrophils significantly increased total MPO activity in the placebo (1.8 times higher than PMA-activated neutrophils) group, while the vitamin C addition significantly decreased the total MPO activity (1.4 times) in the experimental group. Neutrophil activation with PMA significantly increased the percentage of extracellular MPO activity in the placebo group but to a lesser extent than in the experimental group (Figure 1(d)). The addition of vitamin C combined with PMA significantly increased the percentage of extracellular MPO activity in both groups until practically reaching 100% MPO. The distribution of MPO activity between the intra- and extracellular neutrophil medium was strongly influenced by the addition of vitamin C and PMA (Table 3). Extracellular MPO activity increased after neutrophil activation with PMA, both in the placebo and in the experimental groups, and the addition of vitamin C to PMA-activated neutrophils significantly reinforced this response. Intracellular MPO activity significantly increased 4.9 times after neutrophil activation with PMA in the experimental group, whereas the placebo group maintained control values. Vitamin C addition to PMA-activated neutrophils emptied them of MPO, both in the placebo and in the experimental groups.

### 3.3. Influence of PMA, Vitamin C, and DHA Diet Supplementation on Neutrophil Inflammatory Response

Nitrite and nitrate production by immune cells was measured as indicative of NO production related to neutrophil immune toxicity and inflammation activation. We have analysed nitrite and nitrate levels in lysed neutrophils solution (intracellular media) and in free-cells culture supernatants (extracellular media).

**Figure 2:**
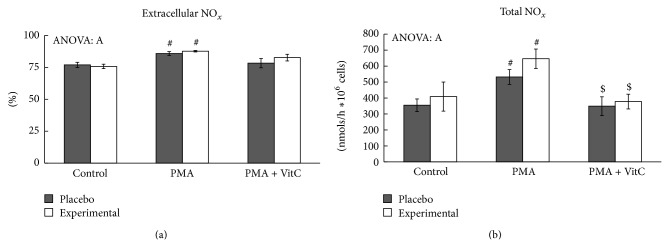
Effects of PMA activation,* in vitro* vitamin C, and DHA diet supplementation on the percentage of extracellular NO_*x*_ and the total NO_*x*_ production. Statistical analysis: two-way ANOVA, *p* < 0.05. S: DHA diet supplementation effect; A: PMA activation effect; S × A: interaction between supplementation and activation effects. # indicates difference with respect to the control group; $ indicates differences between PMA and PMA + VitC group. When interaction S × A exists between supplementation and activation factors, different lowercase letters reveal significant differences. Results are the mean ± SEM.

PMA stimulation significantly increased total NO_*x*_ production in both placebo and experimental groups (Figure 2(a)). Vitamin C addition to PMA-activated neutrophils resulted in total NO_*x*_ production similar to control nonactivated neutrophil values. NO_*x*_ distribution between intracellular and extracellular media was influenced by PMA neutrophil stimulation increasing the percentage in the extracellular media, whereas vitamin C addition to PMA-stimulated neutrophils resulted in NO_*x*_ distribution similar to the control situation. No significant effects were observed in NO_*x*_ production due to DHA diet supplementation. Extracellular nitrate and nitrite concentration demonstrated a similar pattern of change induced by PMA stimulation, vitamin C, and DHA diet supplementation (Table 4). PMA stimulation significantly increased extracellular nitrate and nitrite concentrations in both the placebo and the experimental groups, whereas vitamin C addition to PMA stimulated neutrophils returned to control values. Intracellular nitrate concentration reflected a complementary picture to extracellular nitrate concentration.

The effects of PMA activation, vitamin C, and DHA diet supplementation on the expression of inflammatory genes and cytokine production in neutrophils are shown in Table 5. COX2 expression was significantly increased when neutrophils were stimulated with PMA in the placebo group, and vitamin C addition prevented this increase. An interaction between PMA activation and DHA diet supplementation factors on NF*κβ* expression was observed. NF*κβ* expression maintained control levels in all treatments in the experimental group, whereas it increased in the placebo group after stimulation with PMA but not after the addition of vitamin C. The pattern of IL8 expression was very similar to NF*κβ* expression, with an interaction between neutrophil activation and DHA-diet supplementation factors. IL8 expression significantly increased after neutrophil stimulation with PMA only in the placebo group. TNF*α* expression was affected by PMA activation with a significant increase in the expression only in the placebo group. The addition of vitamin C together with PMA maintained TNF*α* expression at control level both in the placebo and in the experimental groups. There were no significant changes in the expression of MPO when neutrophils were stimulated with PMA. However, MPO expression increased significantly (more than 4 times) in the experimental group, but not in the placebo group, after the addition of vitamin C in combination with PMA.

**Figure 3:**
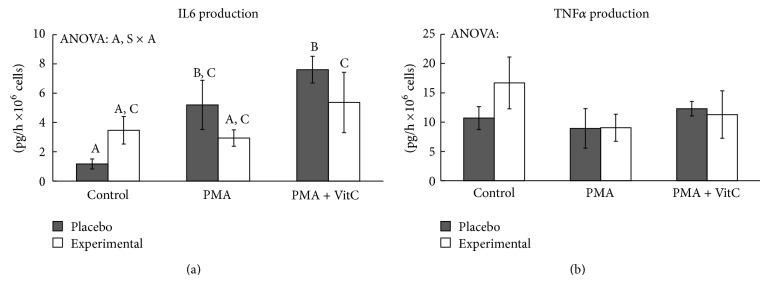
Effects of PMA activation,* in vitro* vitamin C, and DHA diet supplementation on IL6 and TNF*α* production rate. Statistical analysis: two-way ANOVA, *p* < 0.05. S: DHA diet supplementation effect; A: PMA activation effect; S × A: interaction between supplementation and activation effects. When interaction S × A exists between supplementation and activation factors, different lowercase letters reveal significant differences. Results are the mean ± SEM.

We have analysed the production rate of IL6 and TNF*α* (Figure 3). The rate of IL6 production in the extracellular medium increased significantly when neutrophils were stimulated with PMA but was only significant in the placebo group. The addition of vitamin C enhanced the response to PMA activation. No significant differences in the rate of neutrophil TNF*α* production in the extracellular medium were observed.

## 4. Discussion

### 4.1. Assessment of Nutritional Intervention

Diet supplementation for 8 weeks with a DHA-enriched beverage increases DHA content in erythrocytes in accordance with similar nutritional interventions performed in humans [46, 47]. The membrane composition of the placebo and experimental groups' neutrophils should be different in line with the greater DHA content in experimental group's erythrocytes. In fact, similar changes in the fatty acid composition of erythrocyte and leukocyte membranes have been pointed out in child patients with alterative-exudative and allergic inflammation [48], in the same way as diet supplementation with fish oil is known to significantly increase the DHA content of lymphocyte and monocytes membranes [47, 49]. The enrichment of DHA in cellular membranes could modulate several signalling responses to different stimuli [50, 51]. Following fish oil supplementation, omega-3 fatty acids are incorporated into cellular membranes, which can affect lipid-protein interactions and, therefore, the function of embedded proteins [52].

### 4.2. PMA, DHA, and Vitamin C Effects on Oxidative Neutrophil Response

PMA activates the protein kinase signalling pathways in neutrophils [18, 19, 23] and induces neutrophil degranulation [18]. PMA induces higher MPO and CAT activities in extracellular neutrophil media, in accordance with the degranulation effects of PMA [18]. Neutrophils contain several types of exocytosable organelles which hold a battery of molecules that contribute to the precise execution of many neutrophil functions [53]. First, secretory vesicles are mobilized, while tertiary granules, specific granules, and MPO containing azurophilic granules are sequentially mobilized in response to increasingly strong stimuli [53]. The exocytosable CAT is detected in neutrophil organelles [7] but the specific type of organelle is not known. CAT and MPO exit from neutrophils in response to PMA are influenced differently by DHA-diet supplementation and vitamin C addition. DHA diet supplementation enhances exocytosable catalase and also moderates the degranulation of azurophilic MPO containing granules induced by PMA. Vitamin C addition enhances MPO exit but practically prevents (in placebo) or reduces (in the DHA-supplemented group) CAT exit from neutrophils activated by PMA. This may indicate the presence of these two enzymes in different neutrophil organelles. Despite the fact that DHA diet supplementation enhances CAT exit and moderates neutrophil degranulation of azurophilic MPO containing granules induced by PMA, total neutrophil MPO and CAT activities increase in response to PMA in the DHA-supplemented group. DHA supplementation enhances neutrophil oxidative burst by increasing the activity of NAD(P)H-oxidase [54]. The parallelism in the effects of DHA on NAD(P)H-oxidase and CAT activities of PMA-activated neutrophils could be the result of a need to reduce, or to remove, the large amount of ROS produced during the oxidative burst induced by PMA. In addition, MPO gene expression is enhanced by PMA only when a physiological concentration of vitamin C is present, mainly in the DHA-supplemented group. The presence of vitamin C is required to allow complete PMA-induced neutrophil azurophilic granule degranulation until neutrophils are emptied of MPO in response to PMA, whereas DHA diet supplementation enhances PMA-induced CAT exit from neutrophils.

Intense physical activity brings about similar changes in neutrophil CAT and MPO activities as those induced by PMA: decreasing CAT, superoxide dismutase, and glutathione peroxidase activities in neutrophils increasing CAT exit [6, 7] and MPO neutrophil degranulation since increased levels of plasma MPO activity were reported after exercise [50]. Moreover, vitamin C diet supplementation facilitates greater neutrophil activation and antioxidant enzyme secretion to extracellular media induced by intense exercise [4]. We found evidence that vitamin C plays a necessary role in facilitating neutrophil degranulation induced by PMA, in a similar way to that observed in physiological situations such as intense exercise [4, 6, 7, 55].

### 4.3. PMA, DHA, and Vitamin C Effects on Inflammatory Neutrophil Response

Neutrophil capability of producing nitric oxide is stimulated by PMA since total NO_*x*_ levels, markers of NO synthesis, increased after neutrophil activation with PMA. Similarly, it has been pointed out that PMA increases iNOS gene expression in HL60 cells [8], probably as a result of the previous ROS production induced by PMA [8, 24, 56] or due to the presence of inflammatory cytokines such as TNF*α* or IL1 [57]. Vitamin C and DHA diet supplementation influence nitric oxide production and release in response to neutrophil stimulation with PMA. The DHA diet supplemented group had greater intracellular and extracellular nitrate and nitrite levels than placebo group, but the response to PMA stimulation and vitamin C addition was similar to the placebo group. PMA increases the rate of NO production by neutrophils and the extracellular markers of NO synthesis. Neutrophil activation via PMA is driven by protein kinase C isoforms by activating NF*κβ* [15] which regulates the transcription of many acute phase proteins and a large variety of stress response genes, such as inducible nitric oxide synthase (iNOS) and cyclooxygenase-2 (COX2). It also regulates the expression of inflammatory cytokines, chemokines, immunoreceptors, and cell adhesion molecules [58]. The addition of vitamin C at physiological concentration avoids the stimulatory effects of PMA on NO production and NO_*x*_ distribution, maintaining these parameters at non-PMA-stimulated neutrophil level. In this way it has been pointed out that millimolar concentrations of ascorbate inhibit NF*κβ* activation in endothelial cells [13]. The vitamin C effects could indicate the participation of ROS produced by neutrophil activation with PMA in NO production by neutrophils. In fact, it has been pointed out that ROS mediates the induction of iNOS gene expression [56, 59]. However, DHA diet supplementation does not influence the neutrophil response to PMA on NO synthesis.

PMA activates an inflammatory response in neutrophils reflected by its effects on neutrophil degranulation, inflammatory gene expression, and IL6 production. This inflammatory response by neutrophils is mediated by both DHA diet supplementation and vitamin C, both of which prevent the inductive effects of PMA on IL6 production and inflammatory gene expression. It reinforces the idea that both ROS and NF*κβ* participate in the signalling pathway activated by PMA in neutrophils. Several studies have demonstrated the anti-inflammatory effects of omega-3 fatty acids [50, 60, 61]. DHA diet supplementation prevents the enhanced expression of IL8, TNF*α*, COX2, and NF*κβ* genes induced by PMA. This attenuating neutrophil inflammatory response could be due to direct DHA prevention of NF*κβ* activation and translocation into the nucleus. DHA has been described as preventing I-*κβ* phosphorylation and NF*κβ* activation, thereby also preventing NF*κβ* translocation into the nucleus [62], which is required to activate gene expression. Therefore, the DHA attenuating response of neutrophil stimulation with PMA on the gene expression of IL8, TNF*α*, and COX2 could be mediated by DHA interference with NF*κβ* signalling [58]. COX2 [63], IL8, and TNF*α* gene expression [64] are also mediated by activation and internalization into the nucleus of the NF*κβ* pathway which also enables the expression of more than 150 genes [58].

Vitamin C influences the expression of inflammatory genes induced by PMA-activated neutrophils because vitamin C attenuates increased expression of COX2, IL8, TNF*α*, and NF*κβ* in response to PMA. This reinforces the idea that PMA-induced ROS production mediates the activation and translocation of NF*κβ* into the nucleus and the induction of inflammatory genes into neutrophils [13, 64]. The presence of both vitamin C and DHA diet supplementation hinders PMA effects on inflammatory gene expression, but these two factors together do not produce additional effects on the expression of COX2, NF*κβ*, IL8, and TNF*α*. However, MPO gene expression follows a different pattern of change in response to PMA, DHA diet supplementation, and vitamin C. The presence of vitamin C at a physiological concentration only induces increased expression of the MPO gene in the DHA-supplemented group, but not in the placebo group. This different pattern of response is in accordance with the role of vitamin C in the neutrophil degranulation of azurophilic granules indicated above. It suggests the participation of different signalling pathways regulating PMA-induced neutrophil degranulation.

In conclusion, DHA diet supplementation enhances neutrophil antioxidant capability and attenuates inflammatory response after PMA activation. DHA enhances CAT and MPO activities, releases CAT, and moderates degranulation of azurophilic granules containing MPO after PMA activation of neutrophils. Vitamin C facilitates the azurophilic degranulation in PMA-activated neutrophils. Vitamin C and DHA diet supplementation prevent PMA effects on inflammatory gene expression in neutrophils, but these two factors together do not produce additional effects on the expression of inflammatory genes.

## Figures and Tables

**Table 1:** Anthropometric and physical activity characteristics of subjects.

	Placebo	Experimental
Age (years)	19.3 ± 0.4	20.4 ± 0.5
Weight (kg)	76.5 ± 1.8	76.4 ± 3.5
Height (cm)	179 ± 2	180 ± 3
Body mass index (BMI, kg/m^2^)	24.0 ± 0.6	23.5 ± 0.5
Waist-hip ratio (WHR)	0.805 ± 0.012	0.814 ± 0.012
Fat mass (Yuhasz, %)	7.53 ± 0.24	7.21 ± 0.25
Fat-free mass (%)	92.5 ± 0.2	92.8 ± 0.3
Intense physical activity time (min/day)	96.4 ± 57.9	50.4 ± 13.1
Moderate physical activity time (min/day)	68.6 ± 17.1	63.2 ± 14.6
VO_2_max	60.4 ± 18	62.0 ± 0.9
Neutrophils (10^6^ cells/mL blood)	3.47 ± 0.53	2.80 ± 0.12

Statistical analysis: Student's *t*-test for unpaired data, *p* < 0.05.

**Table 2:** Primer sequences and conditions.

Gene	Primer	Conditions	
18S	Fw: 5′-ATG TGA AGT CAC TGT GCC AG-3′	95°C	10 s
	Rv: 5′-GTG TAA TCC GTC TCC ACA GA-3′	60°C	10 s
		72°C	12 s

NF*κβ*	Fw: 5′-AAACACTGTGAGGATGGGATCTG-3′	95°C	10 s
	Rv: 5′-CGAAGCCGACCACCATGT-3′	60°C	10 s
		72°C	15 s

TNF*α*	Fw: 5′-CCCAGGCAGTCAGATCATCTTCTCGGAA-3′	95°C	10 s
	Rv: 5′-CTGGTTATCTCTCAGCTCCACGCCATT-3′	63°C	10 s
		72°C	15 s

IL8	Fw: 5′-GCTCTGTGTGAAGGTGCAGTTTTGCCAA-3′	94°C	10 s
	Rv: 5′-GGCGCAGTGTGGTCCACTCTCAAT-3′	63°C	10 s
		72°C	15 s

MPO	Fw: 5′-TGAACATGGGGAGTGTTTCA-3′	95°C	5 s
	Rv: 5′-CCAGCTCTGCTAACCAGGAC-3′	60°C	7 s
		72°C	10 s

COX2	Fw: 5′-TTGCCTGGCAGGGTTGCTGGTGGTA-3′	95°C	10 s
	Rv: 5′-CATCTGCCTGCTCTGGTCAATGGAA-3′	63°C	10 s
		72°C	15 s

**Table 3:** Effects of PMA activation, *in vitro* vitamin C, and DHA diet supplementation on the distribution of catalase (CAT) and myeloperoxidase (MPO) activities between intracellular and extracellular neutrophil compartments.

		Control	PMA	PMA + VitC	S	A	S × A
Extracellular activity							
CAT (K/10^9^ cells)	Placebo	16.2 ± 1.9	39.4 ± 8.1^#^	46.1 ± 7.3^#^	X	X	
	Experimental	10.3 ± 3.2	112 ± 31^#∗^	90.6 ± 17.8^#^			
MPO (nkat/10^9^ cells)	Placebo	9.18 ± 0.86	34.3 ± 3.8^#^	135 ± 25^#$^		X	
	Experimental	6.94 ± 1.07	40.3 ± 4.3^#^	84.5 ± 33.6^#$^			

Intracellular activity							
CAT (K/10^9^ cells)	Placebo	62.1 ± 12.9	28.2 ± 6.0^#^	77.2 ± 7.29^$^		X	
	Experimental	67.2 ± 6.26	21.6 ± 3.0^#^	68.8 ± 11.9^$^			
MPO (nkat/10^9^ cells)	Placebo	31.5 ± 11.8^a^	38.1 ± 8.93^a^	1.19 ± 0.06^c^		X	X
	Experimental	16.9 ± 2.4^ac^	83.6 ± 18.5^b^	1.23 ± 0.06^c^			

Statistical analysis: two-way ANOVA, *p* < 0.05. S: supplementation effect; A: activation effect; S × A: interaction between supplementation and activation effects. X indicates significant effect of the statistical factor S, A, or S × A. **∗**Differences between placebo and experimental groups; ^#^difference with respect to the control group; ^$^differences between PMA and PMA + VitC group. When interaction S × A exists between supplementation and activation factors, different letters reveal significant differences. Results are the mean ± SEM.

**Table 4:** Effects of PMA activation, *in vitro* vitamin C, and DHA diet supplementation on nitrite and nitrate distribution between extracellular and intracellular neutrophil compartments.

		Control	PMA	PMA + VitC	ANOVA		
					S	A	S × A
Extracellular							
Nitrate (mM)	Placebo	1.07 ± 0.05	1.23 ± 0.04^#^	1.03 ± 0.07^#$^	X	X
	Experimental	1.16 ± 0.10	1.41 ± 0.06^∗#^	1.10 ± 0.07^#$^		
Nitrite (nM)	Placebo	458 ± 54	648 ± 48^#^	422 ± 68^#$^	X	X
	Experimental	545 ± 104	798 ± 65^∗#^	495 ± 69^#$^		

Intracellular							
Nitrate (mM)	Placebo	572 ± 60	447 ± 45	441 ± 65		X	
	Experimental	715 ± 119	494 ± 78^#^	437 ± 60^#^			

Intracellular/extracellular relationships							
Nitrate ratio	Placebo	1366 ± 213	712 ± 77^#^	1305 ± 284^$^		X	
	Experimental	1363 ± 130	646 ± 87^#^	954 ± 152^#^			

Statistical analysis: two-way ANOVA, *p* < 0.05. S: supplementation effect; A: activation effect; S × A: interaction between supplementation and activation effects. X indicates significant effect of the statistical factor S, A, or S × A. **∗**Differences between placebo and experimental groups; ^#^difference with respect to the control group; ^$^differences between PMA and PMA + VitC group. When interaction S × A exists between supplementation and activation factors, different letters reveal significant differences. Results are the mean ± SEM.

**Table 5:** Effects of PMA activation, *in vitro* vitamin C, and DHA diet supplementation on inflammatory gene expression.

		Control	PMA	PMA + VitC	ANOVA		
					S	A	S × A
Gene expression							
COX2	Placebo	1.00 ± 0.12	4.37 ± 1.65^#^	1.21 ± 0.31^$^		X
	Experimental	1.14 ± 0.25	2.32 ± 0.81	1.54 ± 0.33		
NF*κβ*	Placebo	1.0 ± 0.14^a^	1.91 ± 0.34^b^	0.88 ± 0.09^a^	X	X	X
	Experimental	0.90 ± 0.08^a^	0.98 ± 0.14^a^	0.84 ± 0.06^a^			
IL8	Placebo	1.00 ± 0.15*ª*	3.19 ± 1.19^b^	1.01 ± 0.36^a^			X
	Experimental	0.89 ± 0.13^a^	0.89 ± 0.12^a^	1.09 ± 0.18^a^			
TNF*α*	Placebo	1.00 ± 0.12	2.30 ± 0.51^#^	0.99 ± 0.14		X
	Experimental	1.03 ± 0.15	1.24 ± 0.26	0.97 ± 0.12^$^		
MPO	Placebo	1.00 ± 0.16	1.32 ± 0.27	1.73 ± 0.59	X	X
	Experimental	2.09 ± 0.61	1.86 ± 0.66	4.72 ± 1.23^#$^		

Statistical analysis: two-way ANOVA, *p* < 0.05. S: supplementation effect; A: activation effect; S × A: interaction between supplementation and activation effects. X indicates significant effect of the statistical factor S, A, or S × A. ^#^Difference with respect to the control group; ^$^differences between PMA and PMA + VitC group. When interaction S × A exists between supplementation and activation factors, different letters reveal significant differences. Results are the mean ± SEM.
